# Up‐regulation of miR‐10b‐3p promotes the progression of hepatocellular carcinoma cells via targeting *CMTM5*

**DOI:** 10.1111/jcmm.13620

**Published:** 2018-04-24

**Authors:** Lianyue Guan, Degang Ji, Na Liang, Shuo Li, Baozhen Sun

**Affiliations:** ^1^ Department of Hepatobiliary and Pancreas Surgery China‐Japan Union Hospital of Jilin University Changchun Jilin China; ^2^ Teacher's Office of Clinical & Medical Nursing Changchun Medical College Changchun Jilin China

**Keywords:** *CMTM5*, hepatocellular carcinoma, miR‐10b‐3p

## Abstract

In this study, we investigated how miR‐10b‐3p regulated the proliferation, migration, invasion in hepatocellular carcinoma (HCC) at both in vitro and in vivo levels. *CMTM5* was among the differentially expressed genes (data from TCGA). The expression of miR‐10b‐3p and CMTM5 was detected by qRT‐PCR and Western blot (WB). TargetScan was used to acquire the binding sites. Dual‐luciferase reporter gene assay was used to verify the direct target relationship between miR‐10b‐3p and *CMTM5*. WB analysis proved that miR‐10b‐3p suppressed *CMTM5* expression. Furthermore, proliferation, invasion and migration of HCC cells were measured by MTT assay, colony formation assay, transwell assay and wound‐healing assay, respectively. Kaplan‐Meier plotter valued the overall survival of *CMTM5*. Finally, xenograft assay was also conducted to verify the effects of miR‐10b‐3p/*CMTM5* axis in vivo. Up‐regulation of miR‐10b‐3p and down‐regulation of *CMTM5* were detected in HCC tissues and cell lines. *CMTM5* was verified as a target gene of miR‐10b‐3p. The overexpression of *CMTM5* contributed to the suppression of the proliferative, migratory and invasive abilities of HCC cells. Moreover, the up‐regulation of miR‐10b‐3p and down‐regulation of *CMTM5* were observed to be associated with worse overall survival. Lastly, we have confirmed the carcinogenesis‐related roles of miR‐10b‐3p and *CMTM5* in vivo. We concluded that the up‐regulation of miR‐10b‐3p promoted the progression of HCC cells via targeting *CMTM5*.

## 1 INTRODUCTION

As one of the most prevalent malignancies worldwide, hepatocellular carcinoma (HCC) is also the leading cause of cancer‐associated mortality.^1^ The intractable disease evolves more sophisticated due to the dysregulation of different genes by promoting the development and progression of HCC.^2^ Although improvements have been made in surgery and other treatment methods, it remains at a low level in terms of the 5‐year overall survival rate of patients with HCC. The lack of accurate and non‐invasive diagnostic tools makes early diagnosis and remission of HCC difficult, resulting in poor prognosis.^3^ Hence, it is critical to define the mechanisms of hepatocarcinogenesis at molecular level and to develop novel strategies for HCC early diagnosis and prognosis prediction.

MicroRNAs (miRNAs) play an important role in malignancy by targeting various tumour suppressors and oncogenes. They also take part in cancer stem cell biology, angiogenesis and epithelial‐mesenchymal transition, which could also influence carcinogenesis.^4^ Recent emerging evidence has revealed that miR‐10b was up‐regulated in many types of human cancers, such as nasopharyngeal carcinoma, pancreatic cancer, malignant glioma and HCC.^5^, ^6^, ^7^, ^8^ Furthermore, overexpression of miR‐10b‐3p was observed in tumorous tissue specimens of HCC compared with non‐tumour adjacent tissues.^9^ The patients with higher miR‐10b levels showed shorter survival time.^10^ These findings suggest the possible oncogenic role of miR‐10b‐3p in HCC. However, the molecular mechanism remains largely unknown. Given the overexpression of miR‐10b‐3p in cancer tissues, further investigation into the role and molecular mechanisms of miR‐10b‐3p in cancer development and progression was needed.

CMTM5, also called CKLF‐like MARVEL transmembrane domain containing member 5, is a member of *CMTM* family that could inhibit tumour growth.^11^ Being broadly expressed in human tissues, *CMTM5* is usually down‐regulated in carcinoma tissues.^12^, ^13^ Restoration of *CMTM5* may contribute to better morphological transformation, but the antitumour mechanism remains unclear.^14^ It was proposed that the understanding of the antitumour mechanism of *CMTM5* was critical before it became a new target in the gene therapies for tumours.

Previously, few studies have been performed to investigate the correlation between miRNAs and *CMTM*s. Our findings indicated that miR‐10b‐3p directly targeted *CMTM5* and negatively regulated its expression, which therefore inhibited the proliferation, migration and invasion of HCC cells. These results provided new insights into the mechanisms by which miR‐10b‐3p modulated the development of HCC by interacting with *CMTM5*.

## 2 MATERIALS AND METHODS

### 2.1 Cell lines and tissue specimens

Human HCC cell lines HepG2, HCCLM7, Huh‐7 and HLE as well as human normal hepatic cell lines HL‐7702 and THLE3 were purchased from BeNa Culture Collection (Beijing, China) and maintained in Roswell Park Memorial Institute (RPMI) 1640 medium (Sigma, St. Louis, MO, USA) with 90% DMEM+10%FBS (Invitrogen, Gaithersburg, MD, USA) in an atmosphere with 95% air, 5% CO_2_ at 37°C. Tissue specimens of HCC and adjacent non‐tumour tissues (n = 30) were obtained from China‐Japan Union Hospital of Jilin University. Written informed consent was accessed from all patients (n = 350). This study was also approved by the ethics committee of China‐Japan Union Hospital of Jilin University.

### 2.2 Cell transfection

Cell transfection was conducted using Lipofectamine 3000 reagent. HepG2 cells were cultured under normal conditions and were inoculated uniformly to 96‐well plates at appropriate concentrations (approximately 3 × 10^5 ^cells/mL in this case). After adherent cell culture, cell transfection was conducted to miR‐10b‐3p mimics, inhibitors and CMTM5 overexpression plasmids. The cells of the normal group were treated with Lipofectamine only. The mimics and inhibitor etc. were diluted in MEM medium without serum, and then the Lipofectamine 3000 reagent was added to the medium. After incubation for 5 minutes, the diluted Lipofectamine 3000 was mixed with the mixture of last step, which was added to the culture plate of HepG2 cells and incubated at 37°C for 5 hours. Thereafter, the mixture was incubated in MEM medium with 10% FBS for another 48 hours.

### 2.3 Microarray analysis

The microarray data obtained from TCGA Database (https://cancergenome.nih.gov/) was used to screen out the differentially expressed genes in HCC. The data included 18 samples (9 tumour tissues and 9 adjacent non‐tumorous tissues). Affy and limma packages (R packages) were used to read the expression measures and screen out differently expressed genes that were with fold change value greater than 2 or smaller than ‐2 (*P *<* *.05).

### 2.4 QRT‐PCR

Total RNA was extracted and isolated using TRIzol reagent (Life Technologies, Gaithersburg, MD, USA) and assessed by a NanoDrop ND‐1000 Spectrophotometer (NanoDrop, USA). Reverse transcription was then performed using SuperScript III First‐Strand Synthesis System kit (Invitrogen, Carlsbad, CA, USA) and SoFast EvaGreen Supermix (Bio‐Rad, Hercules, CA, USA). Primer sequences were listed in Table 1. Real‐time PCR was performed using NCode™ VILO™ miRNA cDNA Synthesis Kit (Invitrogen) and EXPRESS SYBR GreenER miRNA qRT‐PCR Kit (Life Technologies).

**Table 1:** Primers for qRT‐PCR

	Forward primer 5′‐3′	Reverse primer 5′‐3′
CMTM5	CTTCCTCACCTCCCACAAG	AGATGGAAACCAGGATGATG
GAPDH	TACTAGCGGTTTTACGGGCG	TCGAACAGGAGGAGCAGAGAGCGA
miR‐10b‐3p	GACAGATTCGATTCTAGGGGAAT	CAGTGCGTGTCGTGGAGT
U6	CTCGCTTCGGCAGCACA	AACGCTTCACGAATTTGCGT

### 2.5 Western blot

Proteins were extracted from the transfected cells using cell lysis buffer. The samples were boiled in 5 × SDS loading buffer for 10 minutes and then loaded onto a 10% SDS‐polyacrylamide gel (Beyotime, Shanghai, China). Following electrophoresis, proteins were transferred onto polyvinylidene difluoride membranes (Bio‐Rad) at 300 mA. Immunoblotting was performed by incubating the membranes in anti‐*CMTM5* (1:500 dilution) (Abcam, Cambridge, MA, USA) overnight. The membranes were washed three times with PBS supplemented with 0.1% Tween and incubated with horseradish peroxidase‐conjugated anti‐goat (Abcam) secondary antibodies for 1 hour. After washing the membranes three times with PBS, we detected the bands using Pierce ECL Western blotting substrate (Pierce, Rockford, IL, USA) and quantified the protein expression using densitometric analyses with Quantity One software, version 4.4.0.

### 2.6 Luciferase reporter assay

A bunch of HepG2 cells were seeded onto 24‐well plates to grow in 5% CO_2_ at 37°C till 70% confluence. Then cells were cotransfected with miR‐10b‐3p mimics or control mimics with wild‐type or mutated‐type 3′UTR of *CMTM5* using Lipofectamine 3000 transfection reagent (Life Technologies, Gaithersburg, MD, USA). Thirty‐six hours after transfection, cells were washed with PBS. Luciferase activity was then determined using the Dual‐Luciferase Reporter Assay System (Promega) and a microplate fluorescence reader (BioTek, Winooski, Vermont, USA).

### 2.7 MTT experiments

Forty‐eight hours after transfection, 100 μL of the MTT solution was added to each well of the culture plate. Thereafter, the plate was maintained in an incubator with 37°C and 5% CO_2_. A total of 100 μL of 20% sodium dodecyl sulphate (SDS) was added to each well. The plates were incubated at 37°C for 24 hours. Lastly, a microplate reader was used to read the OD values at 490 nm. Each group contained 3 replicates, and each experiment was repeated in triplicate.

### 2.8 Colony formation assay

Transfected cells were lysed and then seeded onto 6‐well plates. When colonies were visible after approximately 2 weeks, they were washed with PBS and fixed with 4% paraformaldehyde for 20 minutes before they were stained with GIMSA for 30 minutes. Finally, cells were air‐dried. Colonies were counted under an Olympus CK2 phase‐contrast inverted microscope (Olympus, Tokyo, Japan). We randomly chose 5 fields for observation and repeated the experiment for three times.

### 2.9 Wound‐healing assay

In brief, 1 × 10^5^ transfected cells were plated in 6‐well plates till 90% confluence in DMEM containing FBS. Then, a scratch was created on the surface of every cell layer using 200‐μL pipette tips. Detached cells were washed away with PBS. After the scratch, new medium was used for further culture for another 24 hours. Finally, wound‐healing areas were measured and recorded under a microscope.

### 2.10 Cell invasion assay

Cells were initially starved for 24 hours before the suspension. Then they were lysed and washed with PBS before being resuspended with serum‐free media. 1 × 10^5^ cells were plated in the upper well, and 600 μL of DMEM medium containing 10% FBS was served as the chemoattractant in the lower chambers. After 24 hours, invading cells were fixed with 4% paraformaldehyde, stained with 0.1% crystal violet, air‐dried and photographed using a light microscope. The number was also counted under the microscope as well.

### 2.11 In vivo experiments

We purchased 16 healthy and specific pathogen‐free BALB/C mice (5‐week‐old, female, average body weight of 25 g, purchased from Cavens Lab Animal Co., Ltd.). All the mice were given proper water and food. They were housed in 12‐hour light/dark cycles until experiment. All experiments were in accordance with the guidance of the Institutional Animal Care and Use Committee. HepG2 cells (1 × 10^7^ cells in 300 μL PBS) with different transfection were injected subcutaneously into the dorsal left flank of the mice. Tumour volumes (mm^3^) were estimated by measuring the longest and shortest diameters of the tumours.

When the tumour volume reached about 100 mm^2^, the tumour volume was measured every 5 days using a microcalliper. The tumour volume was calculated as (length × width^2^)/2. 25 days later, the mice were killed, and the volumes of the tumours were determined.

### 2.12 Survival analysis

The survival outcomes of 320 patients were analysed. High level was defined as a fold change value bigger than 2, whereas a low level was defined as a fold change value smaller than ‐2. A total of 320 patients were included in the following study (details of the survival analysis have been given in the main text), and among them, 228 were with high miR‐10b‐3p levels, and 92 were with low miR‐10b‐3p levels; and 218 were with low CMTM5 levels, and 102 were with high CMTM5 levels.

### 2.13 Statistical analysis

The statistical analysis and graphical depiction were performed with GraphPad Prism 6.0 software (San Diego, CA, USA) and the SPSS 16.0 software (SPSS, Chicago, IL, USA). All data from at least three independent experiments were expressed as mean ± SD (standard deviation). Statistical differences were determined by ANOVA or Student's *t* test. A value of *P* < .05 was considered statistically significant.

## 3 RESULTS

### 3.1 *CMTM5* was down‐regulated in HCC tissues

**Figure 1:**
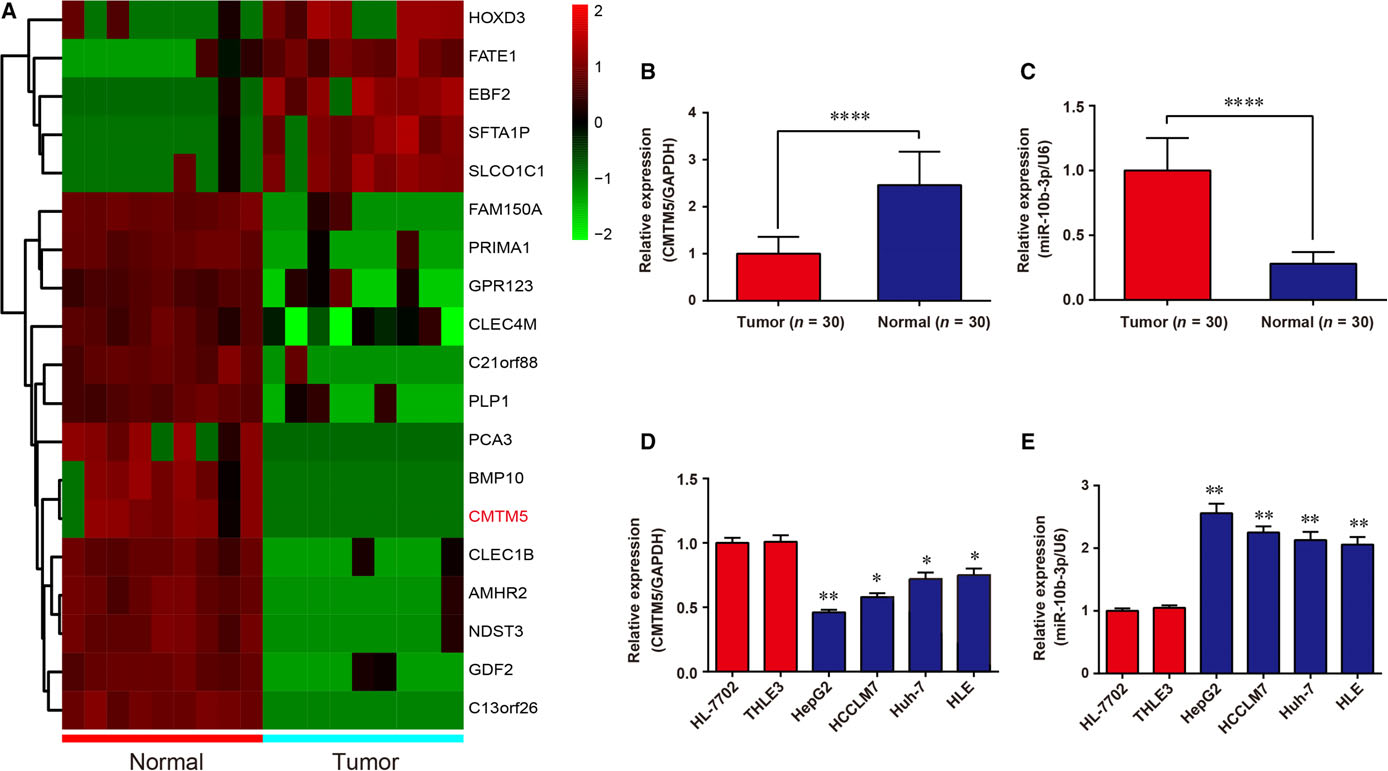
*CMTM5* was low‐expressed, and miR‐10b‐3p was high‐expressed in HCC tissues. A, Heat map showed 5 high‐expressed and 14 low‐expressed genes in tumour tissues (n = 9) and normal tissues (n = 9), with *CMTM5* down‐regulated in HCC tissues. B‐C, QRT‐PCR confirmed that *CMTM5* was low‐expressed and miR‐10b‐3p was high‐expressed in tumour tissues (n = 30). ****P <* *.0001, compared with tumour tissues. D‐E, QRT‐PCR confirmed that *CMTM5* was low‐expressed and miR‐10b‐3p was high‐expressed in HCC cell lines. GAPDH and U6 were used as internal controls for gene and miRNA, respectively. *P <* *.05, **P <* *.01, compared with HL‐7702 cell line

Microarray results demonstrated that 5 genes were up‐regulated, whereas 14 genes were down‐regulated in HCC tissues (Figure 1A), and we chose *CMTM5* for further study. Expression levels of *CMTM5* and miR‐10b‐3p in HCC tissues (n = 30) and adjacent normal tissues (n = 30) were detected using qRT‐PCR. The results revealed that *CMTM5* expression was significantly depressed in tissue specimens of HCC compared with the paired normal tissues (Figure 1B, *P* < .0001). In contrast, miR‐10b‐3p expression was dramatically improved in tissue specimens of HCC compared with the paired normal tissues (Figure 1C, *P* < .0001). Similarly, the expressions of *CMTM5* and miR‐10b‐3p in HCC cell lines and normal human hepatic cell lines were determined using qRT‐PCR. The results showed that *CMTM5* expression was significantly depressed, whereas miR‐10b‐3p was significantly up‐regulated in HCC cell lines compared with normal controls (Figure 1D,E).

### 3.2 *CMTM5* was a direct target of miR‐10b‐3p in HCC cell lines

**Figure 2:**
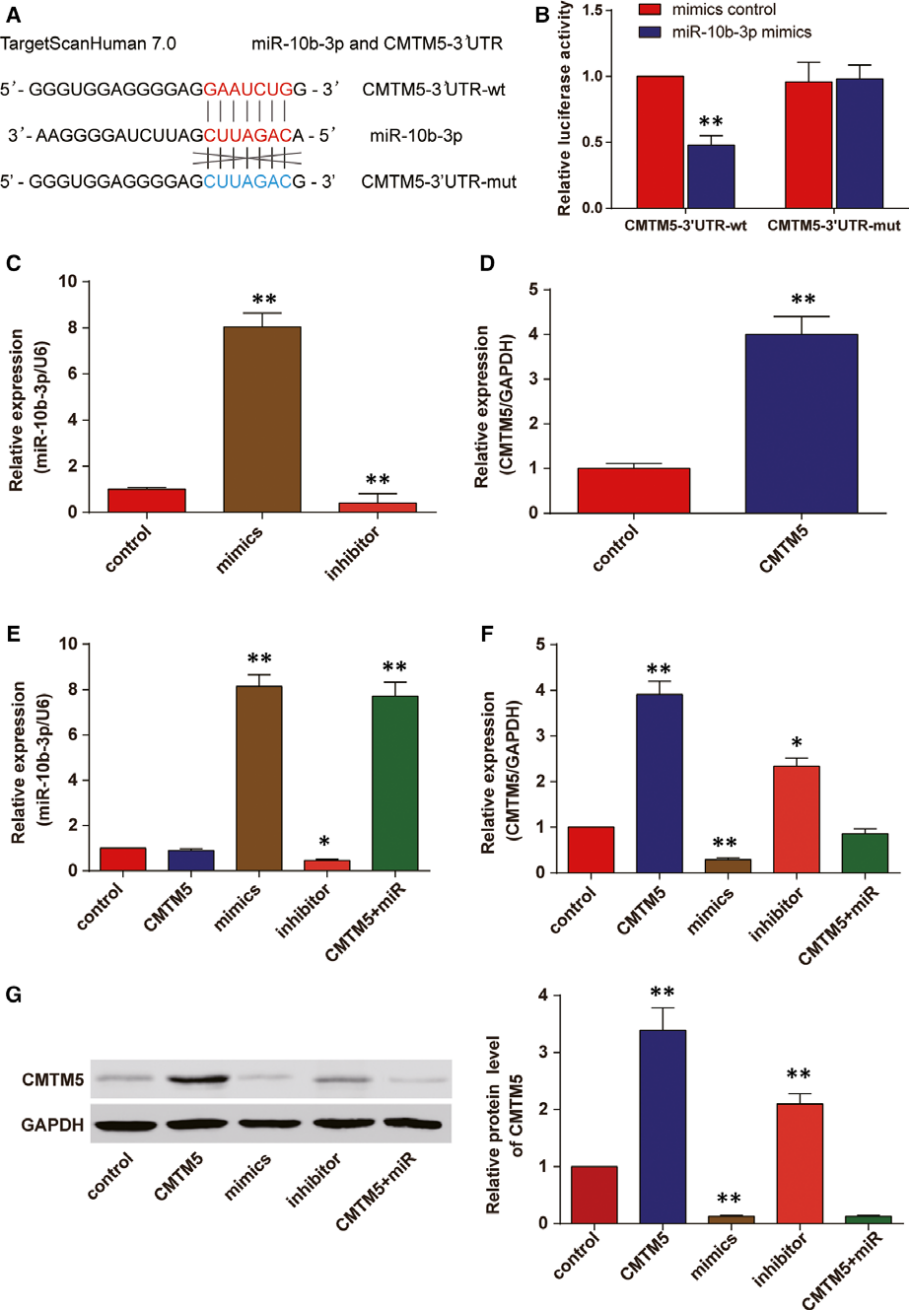
*CMTM5* was a direct target of miR‐10b‐3p in HCC cells. A, The binding sequences of miR‐10b‐3p and *CMTM5* (at position 239‐245) were illustrated. B, Dual‐luciferase reporter gene assay was conducted to verify that miR‐10b‐3p significantly masked the wild‐type 3′UTR but not the mutated‐type 3′UTR of *CMTM5*. **P <* *.01, compared with the mimics control group. C, The transfection efficiency of miR‐10b‐3p mimics and inhibitor in HepG2 cells was confirmed. D, The transfection efficiency of *CMTM5* overexpression plasmids in HepG2 cells was confirmed. E, qRT‐PCR results revealed that mimics group and *CMTM5 *+* *miR group showed significantly up‐regulated miR‐10b‐3p in HepG2 cells. U6 was used as the internal control. F, qRT‐PCR results revealed that cells of *CMTM5* group showed significantly up‐regulated *CMTM5* expression, whereas those of mimics group showed down‐regulated *CMTM5* expression in HepG2 cells. GAPDH was used as the internal control. G, Western blot analysis results confirmed that *CMTM5* was down‐regulated by miR‐10b‐3p mimics in HepG2 cells. *P <* *.05, **P <* *.01, compared with the control group. Mimics: miR‐10b‐3p mimics; CMTM5: CMTM5 overexpression; CMTM5 + miR: CMTM5 overexpression plus miR‐10b‐3p mimics

The binding sequences of *CMTM5* and miR‐10b‐3p were predicted by TargetScan 7.0 (Figure 2A). Luciferase reporter gene assay was conducted to verify the direct target relationship between miR‐10b‐3p and *CMTM5*. The results showed that the luciferase activity in *CMTM5*‐3′UTR‐wt + miR‐10b‐3p mimics group decreased (Figure 2B, *P* < .01). Furthermore, we divided HepG2 cell lines into 5 groups, including CMTM5 group (transfected with *CMTM5* overexpression plasmids), mimics group (transfected with miR‐10b‐3p mimics), inhibitor group (transfected with miR‐10b‐3p inhibitor), *CMTM5 *+* * miR‐10b‐3p group (cotransfected with *CMTM5* overexpression plasmids and miR‐10b‐3p mimics) and control group (transfected with only the transfection reagent). The transfection efficiency of miR‐10b‐3p mimics, inhibitors and *CMTM5* overexpression plasmids was verified (Figure 2C,D). Higher levels of miR‐10b‐3p were seen in mimics and mix (CMTM5 + miR) groups, whereas lower level of miR‐10b‐3p was seen in inhibitor group. The modulation of CMTM5 expression did not affect the expression of miR‐10b‐3p (Figure 2E). On the other hand, the expression of CMTM5 was higher in CMTM5 group and inhibitor group, whereas lower in mimics group. The cotransfection of CMTM5 and mimics did not affect the expression of *CMTM5* (Figure 2F). Western blot results showed that miR‐10b‐3p could suppress *CMTM5* (Figure 2G, *P* < .01). Therefore, we concluded that miR‐10b‐3p was able to suppress *CMTM5* in HCC cell line HepG2.

### 3.3 miR‐10b‐3p promoted HepG2 cell proliferation, migration and invasion by suppressing *CMTM5*

**Figure 3:**
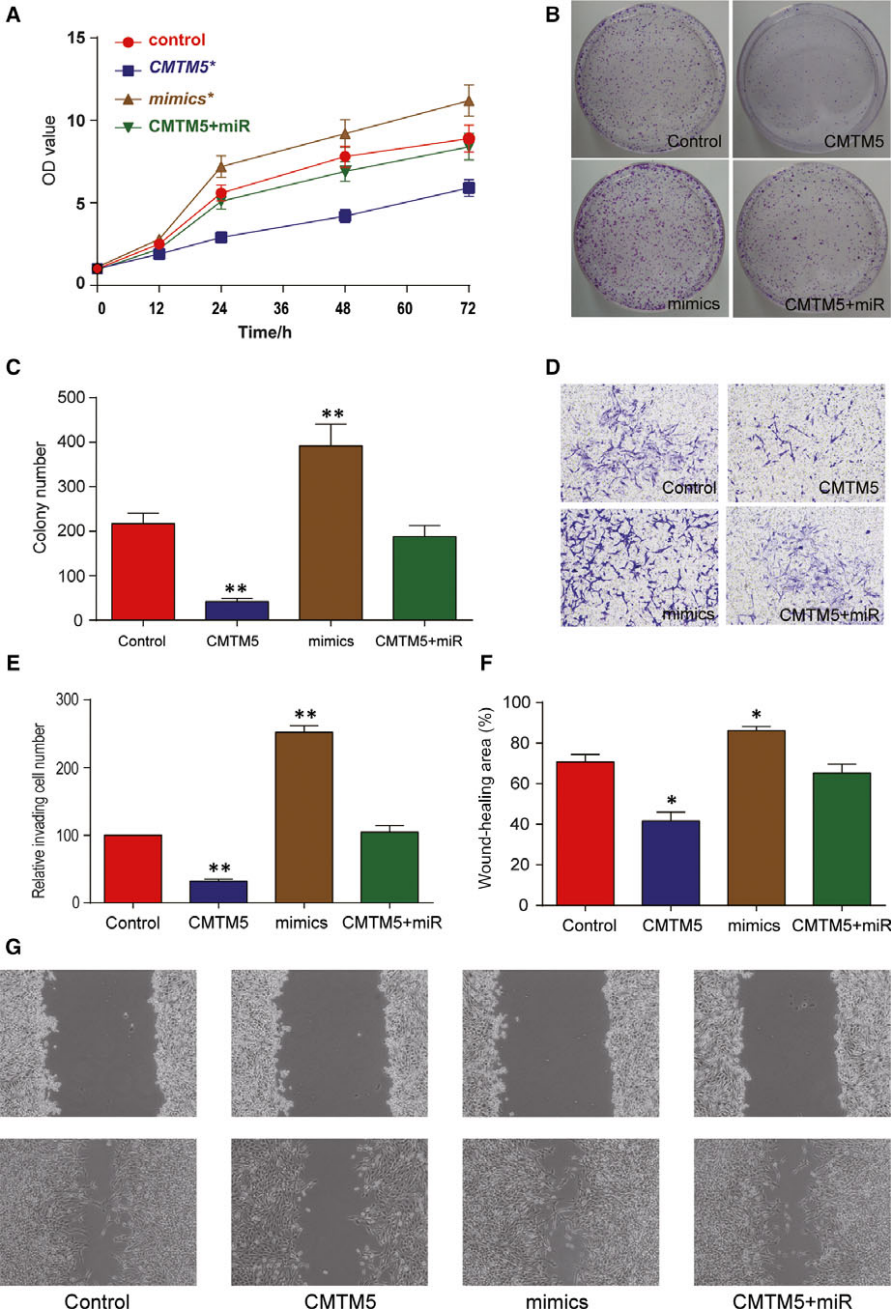
*CMTM5* suppressed HCC cell proliferation, migration and invasion. A, MTT assay results showed that the OD value of HepG2 cells was significantly smaller in CMTM5 group and bigger in mimics group. The OD values of the control group and CMTM5 + miR group did not show significant difference. B, Colony formation assay results showed smaller colony number in CMTM5 group and bigger number in mimics group. C, The histogram of the colony formation assay results. D, Cell invasion assay results revealed that cells of CMTM5 group had smaller invaded number, whereas those of mimics group had bigger number. E, The histogram of the Transwell assay results. F, The histogram of the wound‐healing assay results. G, Wound‐healing assay results demonstrated stronger migration in mimics group and weaker migration in *CMTM5* group. *P <* *.05, **P <* *.01, compared with the control group. Mimics: miR‐10b‐3p mimics; CMTM5: CMTM5 overexpression; CMTM5 + miR: CMTM5 overexpression plus miR‐10b‐3p mimics

Enforced expression of *CMTM5* dramatically reduced cell proliferation (in both MTT assay and colony formation assay) in HepG2 cells, whereas enforced expression of miR‐10b‐3p significantly promoted cell proliferation. The cotransfection of mimics and CMTM5 overexpression plasmids led to no significant change in cell proliferation. (Figure 3A‐C, *P* < .01). Transwell assay results displayed fewer invaded cells in *CMTM5* overexpression group but more in miR‐10b‐3p group (Figure 3D,E, *P* < .01). Besides, the wound‐healing area for *CMTM5* overexpressed cells was smaller, but miR‐10b‐3p‐transfected cells showed bigger wound‐healing area (Figure 3F,G, *P* < .05). These results suggested that *CMTM5* could impair the proliferation, migration and invasion of HepG2 cells.

### 3.4 MiR‐10b‐3p and *CMTM5* predicted overall survival in patients with HCC

**Figure 4:**
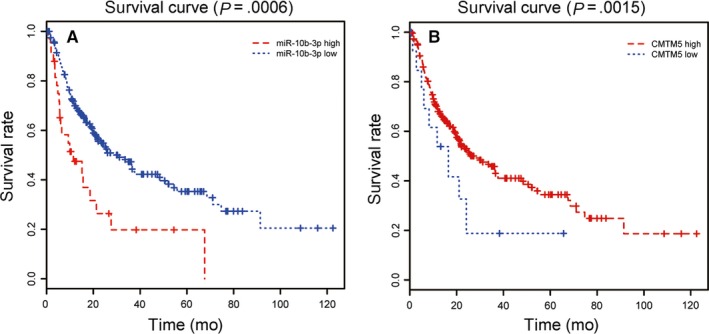
MiR‐10b‐3p and *CMTM5* predicted overall survival outcome of patients with HCC. A, Low expression of miR‐10b‐3p predicted better overall survival outcome, *P *=* *.0006. B, High expression of miR‐10b‐3p predicted better overall survival outcome, *P *=* *.0015

A total of 320 patients were included in the follow‐up study. The overall survival rates of the patients were shown in Figure 4. Patients with low expression of miR‐10b‐3p were accompanied with better overall survival (Figure 4A, *P* = .0006), whereas patients with low expression of *CMTM5* were accompanied with worse overall survival (Figure 4B, *P* = .0015). The results revealed that miR‐10b‐3p had correlation with poor prognosis, whereas *CMTM5* was connected with good prognosis for patients with HCC.

### 3.5 miR‐10b‐3p promoted HCC tumour growth by suppressing *CMTM5* In vivo

**Figure 5:**
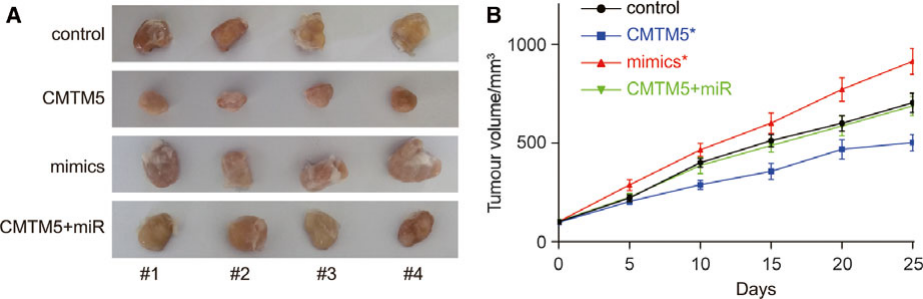
MiR‐10b‐3p promoted HCC tumour growth by suppressing *CMTM5* in vivo. A, Twenty‐five days after the tumour harvest, bigger tumours in mimics group and smaller tumours were seen in CMTM5 group. The cotransfection of mimics and CMTM5 overexpression plasmids did not affect the tumour size. B, The tumour growth was significantly suppressed in CMTM5 overexpressed group but significantly promoted in mimics group. The cotransfection of mimics and CMTM5 overexpression plasmids did not cause the change in tumour growth rate. *P <* *.05, compared with the control group. Mimics: miR‐10b‐3p mimics; CMTM5: CMTM5 overexpression; CMTM5 + miR: CMTM5 overexpression plus miR‐10b‐3p mimics

Twenty‐five days after the tumour harvest, we found bigger tumour sizes in mimics group and smaller tumour sizes in CMTM5 group (Figure 5A). In vivo experiment results suggested that the tumour growth was significantly suppressed in CMTM5 overexpressed group but significantly promoted in mimics group (Figure 5B). The cotransfection of mimics and CMTM5 overexpression plasmids caused no change in tumour growth. The results suggested that miR‐10b‐3p could promote tumour growth by suppressing *CMTM5* In vivo.

## 4 DISCUSSION

In this study, we have conducted a series of studies including microarray assay, in vitro and In vivo assays and identified a novel miR‐10b‐3p/*CMTM5* signalling axis that regulates HCC cell activities. The results revealed that ectopic expression of miR‐10b‐3p had a significant effect on the down‐regulation of *CMTM5* and further influenced biological functions of HCC cells.

Multiple researches showed high level of miR‐10b‐3p expression was detected in HCC tissues and cell lines,^5^, ^15^, ^16^ whereas *CMTM* expression was down‐regulated in liver cancer tissues compared with the adjacent non‐tumour tissues.^11^, ^17^ Similarly, our results also demonstrated high expression of miR‐10b‐3p and low expression of *CMTM5* in HCC cell lines. The opposite expression patterns of miR‐10b‐3p and *CMTM5* in HCC as well as the prediction of Targetscan Human algorithm suggest a potential target relationship between the two. An earlier study revealed that miR‐10b might target *RhoC*,* uPAR*,* MMP‐2* and *MMP‐9*.^15^ Other researches implied *CSMD1* and *CADM1* could also be regulated by miR‐10b.^5^, ^18^ Most of these genes acted as tumour suppressors, so we assumed that miR‐10b could suppress certain anticancer factors including *CMTM5*. In our study, we proved that *CMTM5* was a target gene of miR‐10b‐3p in HepG2 cell line.

The ectopic gene expression could influence cell activities. After we modified the expression of miR‐10b‐3p and *CMTM5*, we found the altered cell proliferation, invasion and migration in single‐transfected group, whereas no obvious alteration on cell activities was found.

As we have found that the modification of miR‐10b‐3p and *CMTM5* expressions changed cell activities, we then supposed that they may also be associated with prognosis. Some studies demonstrated that miR‐10b‐3p level was significantly higher in HCC patients with worse overall survival outcome, indicating higher miR‐10b‐3p expression level was an independent predictor of poor prognosis,^10^, ^18^, ^19^ supporting our results that the overexpression of miR‐10b‐3p had poor prognosis in patients with HCC. However, *CMTM5* expression had no significant impact on the prognosis of patients with ovarian cancer by Kaplan‐Meier method,^13^ whereas our study showed that patients with high expression of *CMTM5* had better prognosis outcome. The difference may be caused by the types of cancer and the different sample sizes (46 in their study and 320 in ours).

Limitations still exist in this study. Future studies are required to further clarify whether other factors participated in miR‐10b‐3p/*CMTM5* signalling axis and affected HCC progression. In addition, miR‐10b and *CMTM5* were found correlated with HCC metastasis. Overexpression of *CMTM5* and *CMTM3*, which are two members of *CMTM* family, inhibited tumour progression both in vitro and In vivo.^11^, ^20^ The two studies indicated that miR‐10b and *CMTM5* could also be useful biomarkers for HCC metastasis, which can be further investigated in the following studies.

In conclusion, our study indicated that up‐regulation of miR‐10b‐3p could promote the progression of HCC cells by suppressing *CMTM5* expression. Our findings may cast new light on the novel therapy targets of HCC.

## CONFLICT OF INTEREST

The authors confirm that there are no conflict of interests.

## ETHICS APPROVAL

This study was approved by the ethics committee of China‐Japan Union Hospital of Jilin University and all participants signed the informed consent.

## AUTHOR CONTRIBUTIONS

Lianyue Guan, Degang Ji conceive research and design of the study. Degang Ji, Na Liang analysed and interpreted the data. Na Liang, Shuo Li contributed to statistical analysis. Shuo Li, Baozhen Sun drafted the manuscript. Baozhen Sun, Lianyue Guan critically reviewed the manuscript. All authors approved the final manuscript.
